# Minimally invasive procedure reduces adjacent segment degeneration and disease: New benefit-based global meta-analysis

**DOI:** 10.1371/journal.pone.0171546

**Published:** 2017-02-16

**Authors:** Xiao-Chuan Li, Chun-Ming Huang, Cheng-Fan Zhong, Rong-Wei Liang, Shao-Jian Luo

**Affiliations:** Department of Orthopedic Surgery, Gaozhou People’s Hospital, Guangdong, China; University of Toronto, CANADA

## Abstract

**Objective:**

Adjacent segment pathology (ASP) is a common complication presenting in patients with axial pain and dysfunction, requiring treatment or follow-up surgery. However, whether minimally invasive surgery (MIS), including MIS transforaminal / posterior lumbar interbody fusion (MIS-TLIF/PLIF) decreases the incidence rate of ASP remains unknown. The aim of this meta-analysis was to compare the incidence rate of ASP in patients undergoing MIS versus open procedures.

**Methods:**

This systematic review was undertaken by following the Preferred Reporting Items for Systematic Reviews and Meta-Analyses Statement. We searched electronic databases, including PubMed, EMBASE, SinoMed, and the Cochrane Library, without language restrictions, to identify clinical trials comparing MIS to open procedures. The results retrieved were last updated on June 15, 2016.

**Results:**

Overall, 9 trials comprising 770 patients were included in the study; the quality of the studies included 4 moderate and 5 low-quality studies. The pooled data analysis demonstrated low heterogeneity between the trials and a significantly lower ASP incidence rate in patients who underwent MIS procedure, compared with those who underwent open procedure (p = 0.0001). Single-level lumbar interbody fusion was performed in 6 trials of 408 patients and we found a lower ASP incidence rate in MIS group, compared with those who underwent open surgery (p = 0.002). Moreover, the pooled data analysis showed a significant reduction in the incidence rate of adjacent segment disease (ASDis) (p = 0.0003) and adjacent segment degeneration (ASDeg) (p = 0.0002) for both procedures, favoring MIS procedure. Subgroup analyses showed no difference in follow-up durations between the procedures (p = 0.93).

**Conclusion:**

Therefore, we conclude that MIS-TLIF/PLIF can reduce the incidence rate of ASDis and ASDeg, compared with open surgery. Although the subgroup analysis did not indicate a difference in follow-up duration between the two procedures, larger-scale, well-designed clinical trials with extensive follow-up are needed to confirm and update the findings of this analysis.

## Introduction

The prevalence of adjacent segment pathology (ASP) requiring additional treatment after spinal fusion surgery has recently become more concerning [1–3]. Adjacent segment degeneration (ASDeg) is represented by radiographic changes in the spine adjacent to the site of spinal fusion surgery, whereas adjacent segment disease (ASDis) is symptomatic deterioration of the adjacent motion segment [4, 5]. The incidence rate of ASDeg 5 years after spinal fusion surgery ranges from 36% to 84% [6], whereas the prevalence of ASDis ranges from 5.2% to 16.5% at 5 years, and 10.6% to 36.1% at 10 years [7, 8]. Pathologically, it is foreseeable that ASDeg develops into ASDis, which leads to axial pain and dysfunction, and eventually results in revision surgery [9]. Several studies have suggested that spinal fusion could accelerate degenerative changes in unfused adjacent segments by increasing adjacent segment motion and placing extra biomechanical stress on intervertebral discs [10, 11]. However, some reports still ascribe this observation to the patients’ propensity for disc degeneration and predisposing risks factors [12–15]. Therefore, based on these data, the etiology of ASDeg and ASDis is most likely multifactorial and remains poorly understood [16, 17].

Although the pathophysiology of these conditions remains uncertain, they have shown to perform a significant impact on modern society, not only physically through increased patient morbidity, but also financially due to loss of productivity and increased healthcare costs [18, 19]. While different, minimally invasive surgery (MIS) should be nearly or exactly as effective as the conventional open technique [20]. The advantage of MIS includes the significant limitation of surgical disruption of soft tissue, such as destruction of paraspinal muscles and ligamentous structures, which may compromise lumbar stability and lead to ASP [21–23]. Minimally invasive transforaminal/ posterior lumbar interbody fusion (MIS-TLIF/PLIF), which has less adjacent tissue destruction and lower morbidity than open surgeries, has been shown to have good long-term clinical outcomes in spinal surgery [24–26].

Hence, whether MIS can decrease the incidence rate of ASDeg and ASDis in patients with degenerative disc disease or spondylolisthesis is unknown. To our knowledge, no meta-analysis has been published on this topic to date. Thus, it is timely to critically review the trials in this field and compare the incidence rate of ASDeg and ASDis among patients in MIS and open procedures.

## Methods

We strictly followed the Cochrane Handbook for Systematic Reviews of Interventions protocol [27]. The study was designed and reported according to the Preferred Reporting Items for Systematic Reviews and Meta-Analyses Statement [28].

### Search strategy

**Fig 1:**
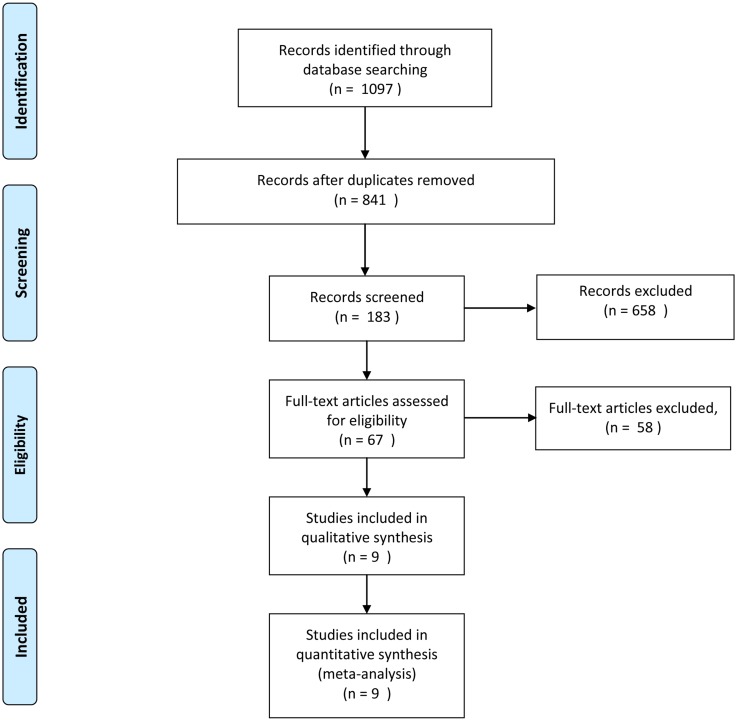
Flow diagram sketches the literatures identified, screened, included and excluded in meta-analysis.

We searched PubMed, EMBASE, SinoMed, and the Cochrane Library databases on June 15, 2016, without restricting the region, publication type, or language. The Mesh terms and Text words were all searched. The related articles function was also used to broaden the search, and the computer search was supplemented with manual searches of the reference lists of all retrieved studies and review articles. The following search strategy was used: (((((ASD) OR ASDis) OR ASDeg) OR ASP) OR adjacent segment disease) OR adjacent segment degeneration) and (((minimally invasive) OR MIS) OR pertacuneous). The detailed search strategy were uploaded in supply materials and flow diagram are shown in Fig 1.

### Eligibility criteria

Two reviewers independently extracted relevant information from each eligible study. Information about the characteristics of the study participants, details of the interventions used, comparisons, and relevant outcomes were recorded. Clinical studies with a randomized controlled trial (RCT) or non-randomized controlled trial (non-RCTs) design in any phase were included. Exclusion criteria included comparative single-arm or no-control trials, case series, case reports, review articles, editorials, letters, surveys, economic studies, and unrelated publications. The outcomes were cross-checked independently, and any inconsistencies in the results were discussed. The exhaustive search is detailed in Table 1.

**Table 1:** The main characteristics of the nine included studies.

Study	Country	Study design	Participants		Matching *	Intervention		Outcomes		Follow-up duration
			Number	Age (yrs)		M	O	M	O	
Yee et al 2014 [21]	USA	Non-RCT	68	M/O: (48±13)/(56±16)	1,2,3,4,5,6,7	52	16	4	3	≥0.5 yrs
Radcliff et al 2014 [22]	USA	Non-RCT	53	Total mean age: (46±9)	1,2,3,4,5,7	23	30	7	9	3.8 yrs
Yu et al 2015 [31]	China	Non-RCT	92	M/O: (51±6)/(53±9)	1,2,3,6	47	45	13	20	5.3 yrs
Tsutsumimoto et al 2013 [32]	Japan	Non-RCT	41	Total mean age: 61	1,2,3,4,5	22	19	3	9	5.7 yrs
Seng et al 2013 [33]	Singapore	Non-RCT	80	M/O: (57±2)/(57±2)	1,2,3,4,5,6,7	40	40	4	4	5 yrs
Parker et al 2014 [34]	USA	Non-RCT	161	NA	1,2,3,6,7	86	75	8	17	5yrs
Ishii et al 2014 [35]	Japan	Non-RCT	78	Total mean age: 62	1,2,3,4,5,6,7	40	38	6	18	46.8mo
Archavlis et al 2013 [36]	Germany	Non-RCT	49	M/O: (67±8)/(68±7)	1,2,3,4,6,7	24	25	1	2	2 yrs
Adogwa et al 2015 [37]	USA	Non-RCT	148	M/O: (57±12)/(56±11)	1,2,3,4,6,7	40	108	0	1	2 yrs

RCT: Randomized controlled trial; NA: not available; yrs: years; mo: months; M: MIS-group; O: Open-group.

Matching

*: 1 = age; 2 = gender; 3 = preoperative diagnosis; 4 = The operation section; 5 = the total fused sites; 6 = operation effects; 7 = other.

### Methodological evaluation and quality assessment

The methodological quality of each study included in the meta-analysis was evaluated using the Cochrane Handbook for Systematic Reviews of Interventions (version 5.2.0). RCTs were evaluated using the Cochrane Collaboration tool to assess the risk for bias, and non-RCTs were assessed using the modified Newcastle-Ottawa scale [29], which consists of 3 factors: patient selection, comparability of the study groups, and assessment of outcomes. A score of 0–9 (recorded as stars) was allocated to each study. Studies with 6 or more stars were considered high quality. The quality of the evidence was assessed according to the guidelines of the Grading of Recommendations, Assessment, Development, and Evaluation working group [30].

### Data analysis and statistical methods

All meta-analyses were performed using Review Manager 5.2.0 (Cochrane Collaboration, UK); publication bias was checked using Stata 11.0. (Stata Corporation, College Station, TX) via the Beg and Egger test [27]. The weighted mean difference and risk ratio (RR) were used to compare continuous and dichotomous variables, respectively. All results were reported with 95% confidence intervals (Cl). Statistical heterogeneity between studies was assessed using the chi-square test. Values of I^2^>50% or P<0.10 indicated heterogeneity between different trials. To demonstrate more robust results, a random-effects model was applied to data analyses.

## Results

The PubMed, EMBASE, SinoMed, and Cochrane Library databases search (Fig 1) yielded 9 studies, including 770 cases, that met the criteria for inclusion [21, 22, 31–37]. Examination of the references cited in these studies and review articles did not yield any further studies.

### Characteristics of eligible studies

The basic characteristics and the matching information of patients clinical characteristics on the 9 trials included in the meta-analysis is respectively summarized in Tables 1 and 2. There were 4 trials from Asia [31–33,35], 1 from Europe [36] and 4 from North America [21,22,34,37]. We also identified and analyzed 6 trials [21,31–33,35,36] that used single-level lumbar interbody fusion. Two trials [36,37] reported short-term incidence of ASP (≤2 years), 2 trials [22,35] mid- term incidence (2–5 years), and 4 trials [31–34] long-term incidence (≥5 years). In addition, 2 studies [32,35] (N = 119) reported an incidence rate for ASDeg and 8 studies [21, 22, 31,33–37] (N = 729) reported the incidence rate of ASDis between 2 groups. In addition, the detailed information of patients clinical characteristics, including diagnosis, involved segments, the fused levels, preoperative scores and postoperative scores, were well matched in all none studies.

**Table 2:** The detailed matching information of patients clinical characteristics.

Study	Diagnosis	Involved segments	The fused levels	Preoperative scores	Postoperative scores
	M/O	M/O	M/O	M/O	M/O
Yee et al 2014 [21]	LDD: 17/2	L1-2: 2/0	Single level: 52/16	NA	NA
	LDH: 7/0	L3-4: 1/1			
	DS: 24/12	L3-4: 4/1			
	LSS: 4/2	L4-5: 27/8			
		L5-S1: 18/6			
Radcliff et al 2014 [22]	LDD: 30/23	NA	Single level: 10/11	NA	NA
			Multilevel: 20/12		
Yu et al 2015 [31]	LDD: 47/45	L3-4: 7/4	Single level:	VAS leg pain: (9.2±1.3)/ (9.7±1.5)	VAS leg pain: (1.7±1.3)/ (1.9±1.5)
		L4-5: 28/26	47/45	VAS back pain: (7.8±0.7)/ (7.8±0.7)	VAS back pain: (1.6±0.8)/ (1.8±1.3)
		L5-S1: 12/15		ODI score: (27.6±2.5)/ (28.1±2.7)	ODI score: (7.2±1.8)/ (6.9±2.1)
Tsutsumimoto et al 2013 [32]	LDD: 22/19	L4-5: 22/19	Single level: 22/19	NA	NA
Seng et al 2013 [33]	LDD: 9/7	L3-4: 2/2	Single level: 40/40	VAS leg pain: (5.9±2.8)/ (5.7±3.2)	VAS leg pain: (0.8±0.4)/ (1.0±0.3)
	DS: 31/33	L4-5: 34/34		VAS back pain: (5.6±3.3)/ (6.2±2.7)	VAS back pain: (1.3±0.4)/ (0.9±0.3)
		L5-S1: 4/4		ODI score: (41.3±20.1)/ (42.1±16.3)	ODI score: (13.6±2.8)/ (12.9±1.9)
				SF-36 MCS: (46.1±11.5)/ (42.6±12.9)	SF-36 MCS: (54.1±13.8)/ (53.3±11.5)
				SF-36 PCS: (34.2±12.5)/ (31.3±8.3)	SF-36 PCS: (47.0±11.0)/ (46.9±10.6)
Parker et al 2014 [34]	LFPs: 86/75	NA	Multilevel: 86/75	Similar clinical presentation	MIS-TLIF accelerated return to work days compared to open-TLIF.
Ishii et al 2014 [35]	DS: 40/38	L4-5: 40/38	Single level: 40/38	JOA scores(NS)	Better improvements in ODI and JOA recovery rate were found in MIS-TLIF.
				ODI (NS)	
Archavlis et al 2013 [36]	DS(grade I): 18/16	L3-4: 2/1	Single level: 24/25	VAS leg pain: 6.7/6.4	VAS leg pain: 2.7/2.6
	DS(grade II): 6/9	L4-5: 16/17		VAS back pain: 6.9/6.6	VAS back pain: 2.5/2.8
		L5-S1: 6/7		ODI score: 46/48	ODI score: 23/24
Adogwa et al 2015 [37]	LDD: 27/81	L1-2: 1/34	Multilevel: 40/108	VAS leg pain: (7.1±3.0)/ (6.6±3.0)	VAS leg pain: (3.8±4.5)/ (2.7±4.1)
	DS: 29/78	L3-4: 7/38		VAS back pain: (7.0±2.5)/ (7.0±2.4)	VAS back pain: (2.4±3.8)/ (2.3±3.7)
		L3-4: 7/41		ODI score: (25.1±8.4)/ (24.6±7.6)	ODI score: (5.8±12.8)/ (7.4±11.0)
		L4-5: 24/83		SF-36 MCS: (41.9±16.7)/ (39.1±18.0)	SF-36 MCS: (4.4±22.7)/ (6.0±22.1)
		L5-S1: 21/62		SF-36 PCS: (24.1±11.3)/ (24.7±9.7)	SF-36 PCS: (8.6±17.7)/ (7.6±15.6)

LDD: Lumbar degenerative diseases; LDH: Lumbar disc herniation; LSS: Lumbar spinal stenosis; DS: Degenerative spondylolisthesis; LFPs: Lumbar spine fusion patients; VAS: Visual analog scale score; ODI: Oswestry disability index; JOA: Japanese Orthopedic Association score; SF-36: 36-Item short form health survey; MCS: Mental component score; PCS: Physical component score; M: MIS-group; O: Open-group; NA: Not available; NS: Not significant.

### Methodological quality of studies included

Although the high methodological quality of the evidence was assessed using the modified Newcastle-Ottawa scale (Table 3), all 9 studies were classified as non-RCTs [21, 22, 31–37]. Therefore, the total risk for bias of the studies included in our meta-analysis was considered low.

**Table 3:** Modified Newcastle-Ottawa Scale (NOS) scores for the included non-RCT studies.

Study	Selection	Comparability	Outcomes	Quality score
Yee et al 2014 [21]	2	3	3	8
Radcliff et al 2014 [22]	2	3	3	8
Yu et al 2015 [31]	1	3	3	7
Tsutsumimoto et al 2013 [32]	2	3	3	8
Seng et al 2013 [33]	1	3	3	7
Parker et al 2014 [34]	2	3	3	8
Ishii et al 2014 [35]	1	3	3	7
Archavlis et al 2013 [36]	2	3	3	8
Adogwa et al 2015 [37]	2	3	3	8

RCT: Randomized controlled trial.

### Quality of evidence

The quality of the evidence for each study was evaluated and is shown in Table 4. Because of a lack of allocation concealment and blinding of participants and personnel, all 9 non-RCTs were downgraded by 2 grades based on the Grading of Recommendations, Assessment, Development, and Evaluation guidelines [30,38]. In addition, the quality of 4 studies [21,32,34,35] was upgraded by 1 grade due to the large effect, whereas the remaining 5 trials [22,31,33,36,37] were neither upgraded or downgraded. Therefore, 4 trials [21,32,34,35]were considered to provide moderate-quality evidence and the other 5 studies [22,31,33,36,37], low-quality evidence.

**Table 4:** Grading of clinical studies following GRADE guidelines.

References	Study design	Risk of bias	Indirectness	Imprecision	Publication bias	Large effect	Plausible residual confounding	Total	Quality of evidence
Yee et al 2014 [21]	Non-RCT	-2	0	0	0	1	0	-1	Moderate
Radcliff et al 2014 [22]	Non-RCT	-2	0	0	0	0	0	-2	Low
Yu et al 2015 [31]	Non-RCT	-2	0	0	0	0	0	-2	Low
Tsutsumimoto et al 2013 [32]	Non-RCT	-2	0	0	0	1	0	-1	Moderate
Seng et al 2013 [33]	Non-RCT	-2	0	0	0	0	0	-2	Low
Parker et al 2014 [34]	Non-RCT	-2	0	0	0	1	0	-1	Moderate
Ishii et al 2014 [35]	Non-RCT	-2	0	0	0	1	0	-1	Moderate
Archavlis et al 2013 [36]	Non-RCT	-2	0	0	0	0	0	-2	Low
Adogwa et al 2015 [37]	Non-RCT	-2	0	0	0	0	0	-2	Low

RCT: Randomized controlled trial.

### ASP incidence rate

**Fig 2:**
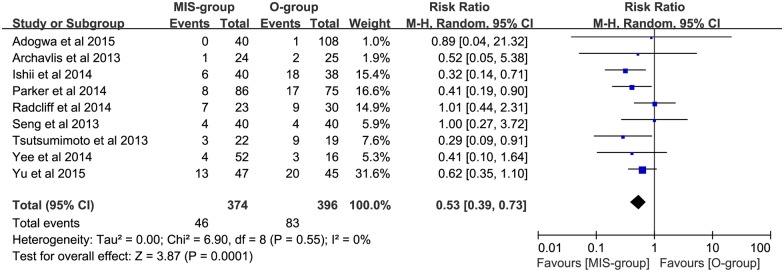
The comparing of ASP incident rate between MIS and open groups.

**Fig 3:**
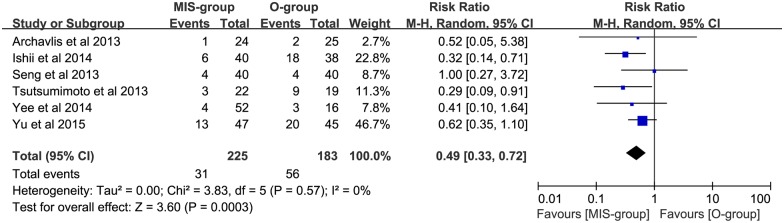
The comparing of ASP incident rate in single level lumbar interbody fusion between MIS and open groups.

**Fig 4:**
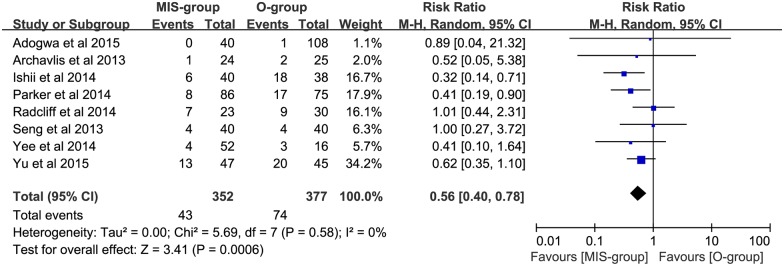
The comparing of symptoms ASDis incident rate between MIS and open grops.

**Fig 5:**
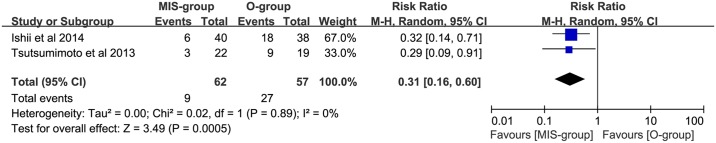
The comparing of radiograph ASDeg incident rate between MIS and open groups.

Pooled data analysis demonstrated low heterogeneity (P = 0.55, I^2^ = 0%) between the 9 trials (N = 770) evaluating the incidence rate of ASP in no less than 6 months; a significantly lower incidence of ASP was seen in the MIS group, compared with the open group (RR: 0.53; 95% CI: 0.39–0.73; P = 0.0001; Fig 2). Of these, 6 trials (N = 408) evaluating single-level lumbar interbody fusion had a lower incidence of ASP in the MIS group (RR: 0.49; 95% CI: 0.33–0.72; P = 0.0003; Fig 3). In addition, 8 trials (N = 729) evaluating the incidence rate of ASDis had a significant reduction in the incidence rate of ASDis in the MIS group (RR: 0.56; 95% CI: 0.40–0.78; P = 0.0006; Fig 4). Two studies (N = 119) evaluating the incidence rate of ASDeg had a decreased incidence rate of ASDeg in the MIS group (RR: 0.31; 95% CI: 0.16–0.60; P = 0.0005; Fig 5).

### Publication bias

**Fig 6:**
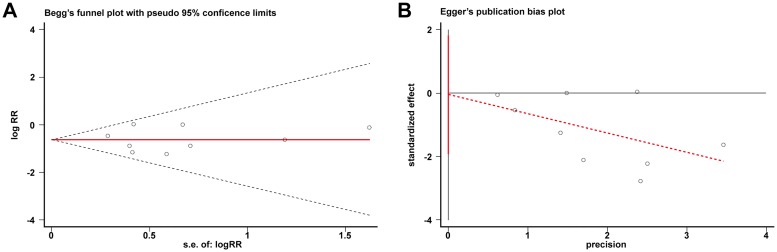
The Beg funnel plot (A) and the Egger funnel plot (B) tests showed no significant publication bias.

We used the Egger and the Beg funnel plots to assess publication bias. We found no evidence of publication bias in either tests (Beg test: P = 0.754, Fig 6A; Egger test: P = 0.958, Fig 6B).

### Subgroup analysis

Subgroup analyses were conducted in different ASP incidence rate follow-up durations. We found no significant difference between trials with low heterogeneity (P = 0.93; I^2^ = 0%). The short-term follow-up incidence rate (≤2 years) reported by 2 studies (n = 197) showed a decreasing trend in the MIS group; the difference was not significant (RR: 0.62; 95% CI: 0.09–4.38; P = 0.63; Table 5). In addition, in the 2 studies (n = 131) with mid-term follow-up (2–5 years) ASP incidence rate, we observed a trend favoring the MIS group; no significant difference was observed between groups (RR: 0.44; 95% CI, 0.09–2.21; P = 0.32; Table 5). Of the 4 studies (n = 374) with long-term follow-up (≥5 years), a significant decrease in the incidence rate of ASP was observed, favoring patients in the MIS group (RR: 0.42; 95% CI: 0.24–0.71; P = 0.001; Table 5).

**Table 5:** The results of different meta-analysis outcomes for MIS-group and Open-group.

Outcomes	Studies	Group number (M/O)	Overall effects		P	Heterogeneity test	
			Effect estimates	95%CI		I^2^(%)	P
ASP incident rate	9	374/396	0.53	0.39, 0.73	0.0001	0	0.55
ASP incident rate (single level fusion)	6	225/183	0.49	0.33, 0.72	0.0003	0	0.57
ASDis	8	352/377	0.56	0.40, 0.78	0.0006	0	0.58
ASDeg	2	62/57	0.31	0.16, 0.60	0.0005	0	0.89
Following-up							
Short term (≤2 years)	2	64/133	0.62	0.09, 4.38	0.63	0	0.78
Middle term (2-5years)	2	63/68	0.44	0.09, 2.21	0.32	76	0.04
Long term (≥5 years)	4	195/179	0.42	0.24, 0.71	0.001	0	0.41

ASP: adjacent segment pathology; ASDis: Adjacent segment disease; ASDeg: Adjacent segment degeneration; M: MIS-group; O: Open-group.

## Discussion

ASDis and ASDeg have become common topic in spine surgery fields due to the obviously increase in fusion surgery in recent years [39]. However, a distinction should be made between ASDis and ASDeg. ASDis is defined as new degenerative changes at a spinal level adjacent to a surgically treated level or levels in the spine, accompanied by related symptoms (radiculopathy, myelopathy, or instability), while ASDeg represents the radiographic changes without the symptomatology [2]. In the current study, the definition of ASDis and ASDeg in all the nine researches included are consistent. Hence, our results about ASDeg and ASDis among the selected papers are credible.

Several publications have compared the incidence rate of ASDeg and ASDis following different treatment interventions [34, 35, 37, 40–42]. However, a meta-analysis including the most recent and relevant data comparing MIS and Open procedures is lacking. Our meta-analysis presents an integrated overview comparing the latest studies on the reduction of incidence rate of ASDeg and ASDis in patients who underwent MIS intervention, compared with those who underwent open procedure. Nine trials comprising 770 patients were included and analyzed. The overall quality of the literature was low including 4 Grade 2 level studies and 5 Grade 3 level evidence. Although the number of studies included in our analysis was small and the data were not sufficient to demonstrate a definite conclusion in all aspects, our findings are supported by the comprehensive evidence of credible outcomes from 770 patients included in the clinical trials. In addition, the detailed information of patients clinical characteristics, including preoperative diagnosis, involved segments, operation sections, preoperative scores and operation effects, showed good matching in all the included nine studies. This matching information may demonstrate the compatibility between the two different surgery procedures. At last, Begg’s and Egger’s funnel plots showed no evidence of publication bias in our meta-analysis, further supporting the credibility of our results.

Based on the data from 9 trials with low heterogeneity, our analysis found a reduction in the incidence rate of ASP in the MIS group (P = 0.0001; Fig 2). The results of single-level fusion between MIS and open groups in lumbar interbody fusion were similar, favoring the MIS group (P = 0.002; Fig 3). In addition, the incidence rate of ASDis (P = 0.0006, Fig 4) and ASDeg (P = 0.0005; Fig 5) both indicated a decreasing trend favoring the MIS group. Moreover, Our conclusion is also heavily supported by the Kaplan—Meier curves analysis, which was conducted in 2 of the studies included in the meta-analysis [21, 32]. It is widely accepted that spine fusion can cause biomechanical changes at adjacent levels, leading to increased range of motion and intradiscal pressure. Correspondingly, the facet joint loading and disc stress were greatly increased and the risk of ASP were highly enhanced. In this study, our result of reduction in both ASDis and ASDeg incidence may be explained by less frequent facet violation in MIS procedure due to the guiding of navigation during the operation process. Besides, compared with open surgery, the lower adjacent tissue destruction associated with MIS surgery may be another protection factor of ASP. Consequently, the lower ASP incidence rate that we found among patients undergoing MIS procedure may reduce adverse outcomes and the need for further surgical intervention.

To date, there are still no long-term follow-up studies evaluating and comparing the incidence rate of ASP between MIS and open procedures. In our subgroup analyses, there was a trend showing MIS-TLIF/PLIF decreased the incidence rate of ASP in all 3 follow-up durations. Although no significant differences were detected in short-term (P = 0.63) and mid-term subgroups (P = 0.32), a significantly lower incidence rate of ASP was observed in the long-term subgroup (P = 0.001). These differences may be due to the small sample size in the short- and mid-term studies. It should be noted that the pathological process of ASDeg and ASDis are considered long-term complications following spinal fusion surgery. Altogether, the trends observed in these subgroup analyses (p = 0.93) may suggest that MIS procedure decreases the incidence rate of ASP in all 3 different follow-up durations.

For further understanding the similar protection or reduction incidence methods of ASP, we also searched for systematic reviews and meta-analyses comparing different interventions of ASP incidence (Table 6). The articles identified mainly focused on motion-preservation procedures and spinal fusion. Of all the 4 systematic reviews or meta-analysis comparing motion-preservation procedures and lumbar spinal fusion in our study [9, 10, 43, 44] 3 of them confirmed the reduction of ASP incidence in patients who underwent the motion-preservation procedure [9, 10, 44], and 1 report was unable to show an association due to limited evidence [43]. Despite these data, none of the studies evaluated MIS versus open procedures suggesting a lack of evidence-based research. In our meta-analysis, the 9 articles were published within the past 3 years, which indicates that evaluating MIS as part of a prospective clinical trial may have added benefits for the patients.

**Table 6:** Systematic review or meta-analysis of ASDis or ASDeg incidence rate between different interventions in lumbar spine surgery.

Author	Year	Publication type	N	n	Patient	Intervention	Outcome
Ren et al [9]	2014	M	13	1270	Lumbar spine surgery	MP (676) and LF (594)	The current evidence suggests that LF **may result in a higher prevalence** of ASDeg or ASDis than MP.
Pan et al [10]	2016	M	15	1474	Lumbar degenerative disease	MP (687) vs LF (787)	The present evidences indicated MP **had an advantage** on reducing ASDeg and ASDis as compared with LF.
Wang et al [43]	2012	S	8	NA	Lumbar spine surgery	MP(NA)vs Spine fusion (NA)	There is **limited evidence** that LF may increase the risk of developing clinical ASP compared **with MP**.
Zhou et al [44]	2013	S	31	NA	Lumbar spine surgery	MP (NA) vs LF (NA)	These results suggested **relative success of the MP** in protecting against ASDeg and ASDis.

MP: Motion-preservation procedures; LF: lumbar spinal fusion; M: Meta-analysis; S: systematic; vs: versus; NA: Not available.

Nonetheless, MIS surgery as a new kind of technology requires a additionally steep learning curve. Besides, this method is associated with significantly longer X-ray exposure dose and need complete protection such as wearing leaded apron and glasses during surgery. Both of this may increase the excess cost. At last, it is a technical challenging of MIS procedure due to smaller operative field that may hinder the accurate decompression, interbody fusion and pedicle screw placement.

We acknowledge that this meta-analysis also had some limitations. First, all 9 studies included were non-RCTs—no RCT were included—which could significantly affect the quality of our meta-analysis. However, conducting RCTs is difficult, because of patient expectations and complex procedures, highlighting the importance of this meta-analysis. Second, small number of studies/patients just including the comparative studies written by MIS-surgeons in this study may weaken our conclusion in certain extent. Consequently, it was difficult to perform a sensitivity analysis with only 9 studies. Third, the inclusion criteria for MIS and open procedures may be different. For example, patients with severe lumbar instability and spondylolysis may have been more likely to be assigned to the open surgery group, which could have affected our results. Lastly, some between-study heterogeneity may be attributable to socioeconomic factors, nutrition, and matching criteria. These differences could have been reduced using a random-effects model, but they would have been difficult to remove all together.

## Conclusion

Based on this meta-analysis, we conclude that patients undergoing MIS procedure may have a lower incidence of ASDeg and ASDis, than those undergoing open surgery. The subgroup analysis evaluating follow-up duration showed no difference between the procedures. Nonetheless, large-volume, well-designed clinical trials with extensive follow-up, are still needed to confirm and update the findings of this analysis.

## Supporting information

S1 File PRISMA checklist. (DOC)
